# Stability indicating green HPLC method for Imeglimin hydrochloride determination in pharmaceutical tablets with comprehensive assessment

**DOI:** 10.1038/s41598-025-32999-4

**Published:** 2026-01-10

**Authors:** Fotouh R. Mansour, Samar H. Elagamy, Almoataz Bellah B. Elbastawissy, Samah F. EL-Malla

**Affiliations:** 1 Department of Pharmaceutical Analytical Chemistry, Faculty of Pharmacy, The medical campus of Tanta University, Elgeish Street, Tanta, 31111 Egypt; 2 Department of Medicinal Chemistry, Faculty of Pharmacy, King Salman International University (KSIU), South Sinai, Egypt

**Keywords:** Imeglimin, Green, Impurity, Forced degradation, In vitro dissolution testing, SIAM, Chemistry, Drug discovery

## Abstract

In this study, an ion pair RP-HPLC method has been developed for separation and quantification of imeglimin hydrochloride (IMG) in the existence of its processing impurity, metformin hydrochloride (MTF). The chromatographic analysis was performed using an XTerra^®^ RP8 column (5 μm, 150 × 4.6 mm) with a mobile phase consisting of a filtered and degassed mixture of 0.1% 1-octane sulfonic acid sodium salt and absolute ethanol (80:20, *%v/v*) at a constant flow rate of 1 mL/min. Detection was performed at 241 nm using a photo diode array (PDA) detector. The method parameters were thoroughly evaluated and validated in accordance with ICH Q2 (R2) guidelines. Linearity was established over the range of 0.4–96.0 µg. mL^− 1^. The use of ion pairing with octane sulfonic acid and ethanol as the mobile phase was effective in achieving an acceptable retention time for the determination of IMG in the presence of metformin and IMG degradation products. Peak purity analysis using PDA detector confirmed that degradation products did not interfere with IMG detection. Furthermore, the method was successfully applied for the accurate quantification of IMG in tablet dosage form, with no interference from excipients, confirming its specificity. Additionally, it was effectively utilized for in vitro dissolution testing of IMG in tablet form, following FDA guidelines. The greenness and applicability of the developed method were assessed using various tools such as the Analytical Eco-Scale, AGREE, MOGAPI, CAFRI, CACI and BAGI demonstrating its environment friendly nature and practicality.

**Supplementary Information:**

The online version contains supplementary material available at 10.1038/s41598-025-32999-4.

## Introduction

Diabetes mellitus (DM) is a chronic metabolic disorder characterized by insufficient insulin action and persistent hyperglycemia, leading to serious macrovascular and microvascular complications. Type 1 DM results from autoimmune destruction of β-cells, whereas type 2 DM arises primarily from insulin resistance linked to lifestyle factors^1–3^. Imeglimin (IMG) received the first approval for use in T2DM in Japan in June 2021. It is the first of a novel class of oral antidiabetic medications called “glimins” which contain tetrahydrotriazine core. It has demonstrated statistically significant glucose-lowering effects and a promising safety and tolerability profile, with no reports of severe hypoglycemia^2–6^.

**Fig. 1:**
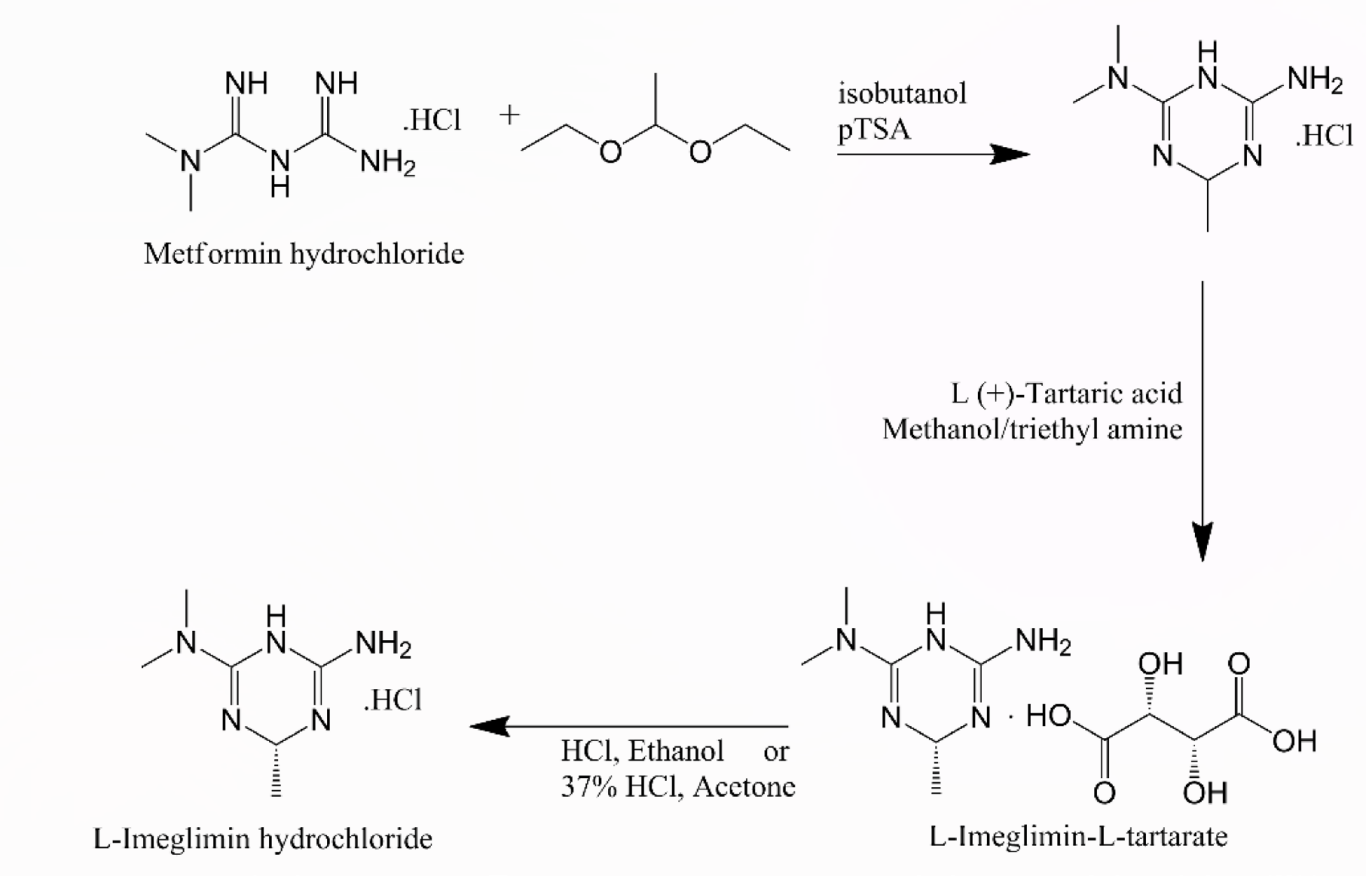
Synthetic pathway L-imeglimin hydrochloride using metformin hydrochloride as starting material.

IMG belongs to the class of organic compounds known as aminotriazines which contain amino group attached to a triazine ring. IMG’s chemical structure is (R)-6-imino-N, N,4- trimethyl-1,4,5,6-tetrahydro-1,3,5-triazin-2-amine hydrochloride, with a chemical formula of C_6_H_14_ClN_5_ and a 191.66 g. mol^− 1^ molecular weight^7^. It has two values of pKa: 17.1 (strongest acidic), and 10.21 (strongest basic), Log P value of -0.92, and its water solubility is 4.63 mg. mL^− 1^^8,9^. Metformin HCl (MTF) is a potential organic related impurity for IMG, which is used as starting material in its synthesis (Fig. 1).

Several analytical methods have been reported for the estimation of IMG, including direct UV spectrophotometry^9,10^ and chromatographic methods^7,8,11–15^. The reported HPLC methods include conventional RP-HPLC techniques developed for the quantification of IMG in tablet dosage forms^11–13^. In addition, stability-indicating methods have been developed using RP-HPLC^7^ and UPLC^13^ equipped with photodiode array (PDA) detectors for detection of degradation products. More advanced stability studies have reported including LC method coupled electrospray ionization/atmospheric pressure chemical ionization mass spectrometry (LC-ESI/APCI-MS)^15^ and LC coupled with quadrupole time-of-flight tandem mass spectrometry (LC-Q-ToF-MS/MS) along with nuclear magnetic resonance (NMR) spectroscopy for impurity profiling and structural elucidation^8^. However, the previously reported RP-HPLC methods suffer from low retention times for IMG, which limits the effective separation and detection of all possible degradants. Furthermore, these methods utilize methanol and acetonitrile which pose significant health and environmental hazards, including toxicity, cytotoxic metabolites, and risks associated with inhalation or skin contact. Additionally, acetonitrile has experienced supply shortages and high market costs due to fluctuations in its industrial production^16,17^. To overcome these concerns, several environmentally friendly solvents have been explored, with ethanol (EtOH) emerging as the most practical green alternative. EtOH offers low toxicity, biodegradability, lower vapor pressure, and reduced environmental impact, and a suitable UV cutoff (≈ 210 nm). EtOH is also more affordable and easier to dispose of than methanol and acetonitrile, making it highly suitable for routine pharmaceutical analysis^18–20^.

Forced degradation studies are essential in analytical method development, exposing the drug to harsher conditions to generate potential degradation products^21,22^. These studies reveal degradation pathways of active pharmaceutical ingredient API and products. Typically, the drug is stressed to achieve 5–20% degradation, providing essential information about its stability under various environmental factors such as heat, light, and humidity^23–28^.

The absorption of a drug substance after oral administration of a solid dosage form depends primarily on its release from the drug product, its dissolution under biological conditions, and its permeability through the gastrointestinal membranes. Given the significance of the first two stages, in vitro dissolution testing plays a crucial role in predicting the in vivo performance of the drug substance. The dissolution media include 0.1 M HCl, acetate buffer (pH 4.5), and phosphate buffer (pH 6.8)^29,30^.

This study aims to develop a novel, green RP-HPLC method for the determination of IMG in tablet dosage forms and for assessment of its dissolution behavior. Notably, this represents the first HPLC method specifically designed to study the dissolution profile of IMG. The novelty of the method also lies in its use of ion-pairing reagent of octane sulfonic acid and ethanol as an environmentally friendly mobile phase. The rationale for employing ion-pair chromatography is that MET and IMG cannot be effectively separated on conventional RP-HPLC columns (C8 or C18) without ion-pairing reagents or specialized stationary phases. For instance, the USP monograph for MET utilizes an L9 cation-exchange column, and the USP assay for MET extended-release tablets depends on sodium 1-heptanesulfonate with a C18 column to achieve adequate retention and separation^31^. This approach achieves improved retention of IMG compared to the reported HPLC methods, which suffer from short retention times that hinder the detection of all degradation products. The proposed method enables effective separation and quantification of IMG in the presence of its organic impurity, MTF, well as stress-induced IMG degradation products, thereby addressing the limitations of existing stability-indicating methods.

## Experimental

### Instrumentation

HPLC Agilent 1260 Infinity Series (Agilent Technologies, Inc. USA) equipped with quaternary pump (G1311C), Autosampler (G1329B), column oven (G1316A), and PDA-Detector (G1315D). For signal monitoring and processing ChemStation software was used. Mixing and dissolving of solutions and dissolution media was done using Stuart magnetic stirrer; US152, UK. Mobile phase degassing was done using Elmasonic; S 300 H, Germany, and a shaking water bath: Memmert; WNB22, Germany was also used. Dissolution testing was done using USP apparatus II (Paddle) dissolution tester: Pharma Test; PT WS100D, Germany.

### Materials and reagents

Imeglimin Hydrochloride (IMG, 99.19% purity) and impurity: Metformin Hydrochloride (MTF, 99.67% purity) were supplied from Metrochem API Pvt Ltd, India. Twymeeg^®^ 500 mg Tablets were provided from Sumitomo Dainippon Pharma Co., Ltd., Japan. EtOH (absolute) HPLC grade was obtained from Merck, Germany. 1-Octane sulfonic acid sodium salt monohydrate was provided from (Scharlau, Spain). The excipients used in the placebo preparation include: hydroxypropyl cellulose (Ashland, USA), Talc (AWA for chemicals, Egypt), croscarmellose sodium (FMC, USA), hypromellose (DuPont, Spain), titanium oxide (Elgomhoreya, Egypt), macrogol 4000 (Pharma egyptia, Egypt), colloidal silicon dioxide (Wacker, Germany), saccharin sodium hydrate (JMC corporation, Korea), and magnesium stearate (Italmatch, Italy). Hydrochloric acid (Scharlau, Spain, 36.5–38%) used in dissolution study, and acidic stress degradation studies was supplied from Scharlau, Spain. Sodium hydroxide pellets (Scharlau, Spain, 98.5%) and hydrogen peroxide (Merck, Germany, 30%) were used for forced degradation study.

Diluent in assay is purified water, while in dissolution is 0.1 M HCl. Dissolution media was prepared according to FDA guidance by mixing 8.5 mL of 36.5–38% hydrochloric acid with 900 mL of purified water and then addition of purified water to 1000 mL.

### Chromatographic conditions

The chromatographic analysis was done using XTerra^®^ RP8 5 μm (150 × 4.6 mm, 5 mm) column with a mobile phase consisting of filtered and degassed mixture of 0.1% of 1-octane sulfonic acid sodium salt monohydrate and absolute ethanol in a ratio of (80:20, % *v/v*). The injection volume was 5 µL, at 1 mL.min^− 1^ flow rate, the column’s temperature was preserved at 40 °C, with detection of drug substance at 241 nm, and the total run time was 8 min. Peaks integration and calculation was done using ChemStation software.

### Standard solutions and calibration curve

#### Standard solutions

##### For assay

The standard stock solution was prepared by weighing 40.0 mg of IMG, then transferred into 100-mL volumetric flask in which it was dissolved in 20 mL of absolute EtOH and 50 mL of purified water with sonication for 5 min with intermittent shaking, then the volume was completed with purified water to have a stock solution of 400.0 µg. mL^-1^ concentration. A working standard solution containing 40.0 µg. mL^-1^ of IMG was prepared by diluting 5.0 mL of the standard stock solution to 50.0 mL with purified water.

Different volumes (0.05-12.0 mL) of IMG standard stock solution (400 µg. mL^− 1^) were accurately transferred into a series of 50-mL volumetric flasks and then were completed with purified water to volume to have solutions ranging from 0.4 to 96.0 µg. mL^− 1^ of IMG. Then the calibration curve was made by calculating the average peak areas (mAU) of three injections of each concentration at 241 nm versus the concentration of IMG (µg. mL^− 1^).

##### For dissolution testing

A standard stock solution of 400.0 µg. mL^− 1^ of IMG was prepared by weighing and dissolving 40.0 mg of IMG in 20 mL absolute EtOH, and 50 mL of 0.1 M HCl in a 100-mL volumetric flask. The solution was sonicated for 5 min with occasional shaking and then diluted to volume with 0.1 M HCl.

Different volumes (1–8 mL) of IMG stock standard solution for dissolution were accurately transferred into a series of 50-mL volumetric flasks and were completed with 0.1 M HCl to volume to have solutions ranging from 8.0 to 64.0 µg. mL^− 1^. The calibration curve was made by calculating the average peak areas (mAU) of three injections of each concentration at 241 nm versus the concentration of IMG (µg. mL^− 1^).

##### For preparation of MTF stock solution

The stock solution of MTF was prepared by weighing and transferring 40.0 mg of MTF into 100-mL volumetric flask, then was dissolved, and volume completed with purified water to obtain MTF stock solution (400.0 µg. mL^− 1^ of MTF).

##### For assessment of system suitability

A solution containing (40.0 µg. mL^-1^ IMG, 4.0 µg. mL^-1^ MTF) was prepared by diluting (5mL) from IMG standard stock solution (400.0 µg. mL^-1^), and (0.5 mL) from MTF stock standard solution (400.0 µg. mL^-1^ of MTF) into a 50-mL volumetric flask, was diluted to volume with purified water. This system suitability solution was used during the method development to choose and optimize the chromatographic conditions to obtain the optimum conditions.

### Specificity

#### Forced degradation studies

IMG was exposed to various stress conditions including oxidative, alkaline, acidic, thermal, and photo-degradation. The concentration of the drug was 40.0 µg. mL^− 1^ in all stress studies. At least two samples were prepared for each stress condition.

The percent of degradation in each stress condition was calculated using the following equation:

$$\% {\text{ of degradation}}\, = \,\frac{{\left( {{\text{~PST}} - {\text{PDEG}}} \right){\text{~}}}}{{{\text{PST}}}}*{\text{1}}00$$

Where: P_ST_ is the average of peak areas of IMG in the solution of standard (no degradation), and P_DEG_ is the average peak areas of IMG in the solution of stress condition.

Stress conditions are stopped in each study type after sufficient degradation occurred (% of degradation reaches or exceeds 20%). The following defines the optimal stress conditions; reagent concentration, temperature applied, and exposure time in each stress condition study:

##### Acid degradation

The acidic stress testing was carried out by transferring 2 mL from 5 M HCl to 5.0 mL of IMG standard stock solution (400.0 µg. mL^− 1^), reflux for 24 h, then neutralize with 2 mL from 5 M NaOH, and then completing the volume with purified water to 50 mL.

##### Base degradation

The basic stress testing was carried out by transferring 2 mL from 2 M NaOH to 5.0 mL of IMG standard stock solution (400.0 µg. mL^− 1^), reflux for 24 h, then neutralize with 2 mL from 2 M HCl and then complete the volume with purified water to 50 mL.

##### Oxidative degradation

Oxidative stress testing was carried out by transferring 2 mL of (30% *v/v*) hydrogen peroxide to 5.0 mL of IMG standard stock solution (400.0 µg. mL^− 1^), reflux for 24 h, and then completing the volume with purified water to 50 mL.

##### Photo degradation

In order to perform photo degradation, 5.0 mL of IMG standard stock solution (400 µg. mL^− 1^) was exposed to UV light providing an overall illumination of 6 million lux hours. Then sample was diluted in 50-mL volumetric flask then completing the volume with purified water.

##### Thermal degradation

To perform thermal degradation, 5.0 mL of IMG standard stock solution (400.0 µg. mL^− 1^) was transferred into a 50-mL volumetric flask, putting it in a water bath that was kept at 80 °C for 360 min, and then completing to volume with purified water at room temperature.

#### Specificity of the dissolution method

Dissolution method’s specificity was evaluated by injecting the placebo, dissolution medium, standard IMG, and test IMG. The placebo was transferred to a vessel containing 500 mL of 0.1 M HCl and rotated at 50 rpm for 30 min at 37 ± 0.5 °C, then after the specified time solution was withdrawn, diluted, filtered and injected on HPLC.

### Application to dosage form assay

Ten Twymeeg^®^ tablets were grinded in a mortar, the powdered tablets were homogenized, and then a quantity equivalent to 500 mg of IMG was transferred into a 250-mL volumetric flask and then dissolved in 50 mL of absolute EtOH and 100 mL of purified water with the aid of sonication for 15 min with intermittent shaking, then was completed with purified water to volume. Stock sample solution is claimed to contain 2000.0 µg. mL^− 1^. Then 2.0 mL was diluted into a 100-mL volumetric flask, and it was completed with purified water to volume. The sample solution is claimed to contain 40.0 µg. mL^− 1^ of IMG. Six tests of the same sample were prepared by the same procedures from the homogenized powdered tablets. Samples were filtered on 0.45 μm Nylon syringe filter prior to injection on HPLC, with the first 3 mL of the filtrate being discarded. The constructed calibration curve was used to calculate the concentration of IMG in the tablets.

### Application to in vitro dissolution testing

#### Dissolution testing conditions

Since there are no specific dissolution conditions mentioned in FDA database for IMG dissolution, the dissolution method was developed according to FDA guidance for immediate-release solid dosage form, using USP apparatus II (paddle). 0.1 M HCl was used as dissolution media with volume of 500 mL per vessel. Rotation speed was set at 50 rpm. The temperature was kept at 37 °C ± 0.5 °C. The time point was 30 min.

#### Sample preparation

Six tablets were tested by adding each tablet into each of six dissolution vessels containing 500 mL of 0.1 M HCl. The theoretical IMG concentration in each dissolution vessel was calculated by dividing the labeled amount of IMG in the tablet in microgram by the dissolution media volume in each dissolution vessel if 100% release occurs.

$$\:Labelled\:IMG\:conc.\:in\:each\:vessel=\frac{500000\:\mu\:g}{500\:mL}=1000\:{\mu\:g.mL}^{-1}$$

Samples of 10.0 mL were manually withdrawn from each vessel after 30 min., filtered against 0.45 μm nylon syringe filter, with the first 3 mL of the filtrate being discarded (stock sample solution for dissolution, 1000 µg. mL^− 1^), then an accurately 2.0 mL of stock sample solution for dissolution was diluted to 50 mL with dissolution media to obtain 40.0 µg.mL^− 1^ solution. All samples were filtered against 0.45 μm nylon syringe filter before HPLC injection. Dissolution media was also injected.

#### Chromatographic conditions for dissolution study

The chromatographic conditions of dissolution were as the same as used under assay ("Chromatographic conditions").

#### Calculations

The regression equation was utilized to calculate the percentage of the drug dissolved at each time point, and the result was determined using the following formula:

$$\:\text{\%}\:\text{r}\text{e}\text{l}\text{e}\text{a}\text{s}\text{e}\:\text{a}\text{t}\:\:\text{c}\text{e}\text{r}\text{t}\text{a}\text{i}\text{n}\:\text{t}\text{i}\text{m}\text{e}\:\text{t}\:=\frac{\text{C}\text{o}\text{n}\text{c}\text{e}\text{n}\text{t}\text{r}\text{a}\text{t}\text{i}\text{o}\text{n}\:\text{o}\text{f}\:\text{d}\text{r}\text{u}\text{g}\:\left({{\upmu\:}\text{g}.\:\:\text{m}\text{L}}^{-1}\right)\:\text{a}\text{t}\:\text{t}\text{i}\text{m}\text{e}\:\text{t}\:\text{x}\:\text{p}\text{o}\text{t}\text{e}\text{n}\text{c}\text{y}\:\text{o}\text{f}\:\text{w}\text{o}\text{r}\text{k}\text{i}\text{n}\text{g}\:\text{s}\text{t}\text{a}\text{n}\text{d}\text{a}\text{r}\text{d}\:\text{i}\text{n}\:\text{p}\text{e}\text{r}\text{c}\text{e}\text{n}\text{t}\text{a}\text{g}\text{e}}{\text{l}\text{a}\text{b}\text{e}\text{l}\text{e}\text{d}\:\text{c}\text{o}\text{n}\text{c}\text{e}\text{n}\text{t}\text{r}\text{a}\text{t}\text{i}\text{o}\text{n}\:\text{o}\text{f}\:\text{s}\text{a}\text{m}\text{p}\text{l}\text{e}\:\text{s}\text{o}\text{l}\text{u}\text{t}\text{i}\text{o}\text{n}}$$

Where the Labeled concentration of sample solution is 40.0 µg. mL^− 1^.

## Results and discussion

IMG is synthesized through a series of reactions using MTF as the starting material. Figure 1 illustrates the steps of the synthetic pathway of IMG from MTF^32^. Therefore, MTF is considered a potential organic impurity.

### Chromatographic conditions optimization

Method optimization was performed using a mixed solution of IMG and MTF to ensure effective separation of the analyte from its impurity with good resolution and within an acceptable run time. As most of the reported methods rely on methanol and acetonitrile solvents known for their environmental and health hazards, this work aimed to develop a greener HPLC method. The initial trial utilized a mobile phase consisting of purified water and ethanol (80:20, %*v/v*) on a C18 column. However, this system resulted in poor retention and interaction of IMG with the reversed-phase stationary phase, leading to co-elution of the IMG peak with the solvent front at approximately 1.5 min (Fig. S1). This inadequate retention was attributed to the high hydrophilicity and low Log P value of IMG. Using buffer solutions to improve retention was considered; however, this approach was not feasible due to the basic nature of IMG. Additionally, HPLC columns tolerate pH 2–7, buffers outside this range could damage the column; therefore, buffer selection alone could not provide an adequate solution. To address this problem, a C8 column (which is less hydrophobic than C18) was used in combination with an ion-pairing reagent to enhance retention and improve peak separation. Since IMG is a salt compound that acquires a positive charge upon ionization, negatively charged ion-pairing reagents were evaluated. Ion-pairing reagents separate analytes via a dynamic ion-exchange process, where the ion pair reagent adsorbs on the column and solutes exchange ions with its counterion^33,34^.

Various ion-pairing reagents, including hexane, heptane, and 1-octane sulfonic acid sodium salts, were evaluated. The use of 1-octane sulfonic acid sodium salt resulted in sufficient retention time and effective separation of IMG from MTF. As previously mentioned, due to the highly hydrophilic nature of IMG, a high percentage of organic solvent is not required for its elution. Different ethanol ratios (15%, 20%, and 25%) were tested. A higher organic content of 25% resulted in shorter retention times (~ 4.5 min), which could compromise the separation and detection of all degradation products. Conversely, 15% ethanol extended the retention time to approximately 8 min but led to an undesirably long overall run time. A mobile phase containing 20% ethanol was found to be optimal, providing sufficient elution of the drug at a retention time of approximately 6.05 min. This condition allowed effective separation from MTF and potential degradation products, while maintaining a reasonable run time suitable for routine analysis.

**Fig. 2:**
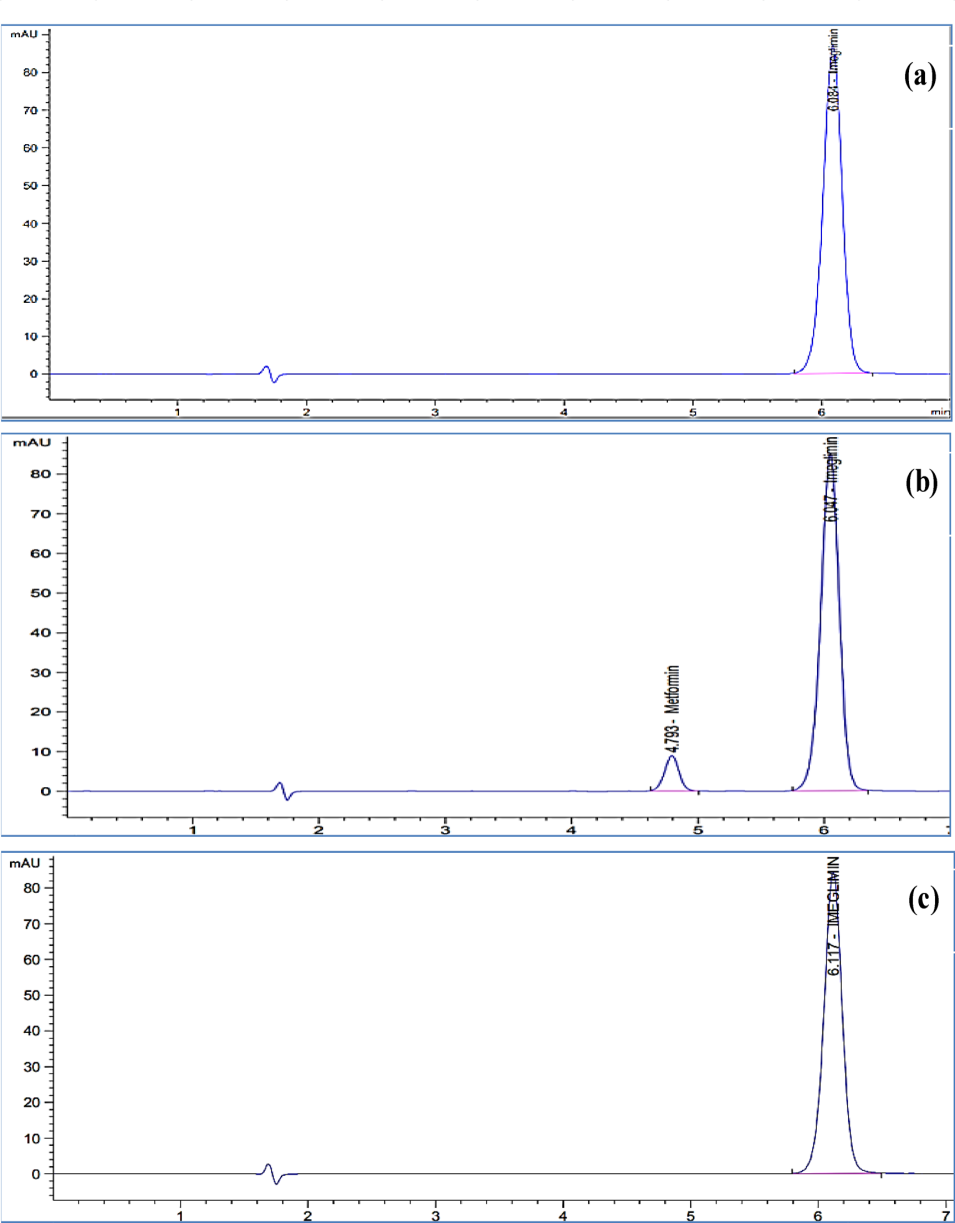
The chromatograms of 40.0 µg. mL^− 1^ IMG at the optimum chromatographic conditions; (**a**) IMG drug substance; (**b**) spiked with impurity MTF (4.0 µg. mL^− 1^); and (**c**) in tablet dosage form.

Different brands of C8 columns were tested. Although they provided acceptable capacity factors, the Luna C8 column resulted in prolonged retention times and extended run times, making it impractical for routine QC analysis. Therefore, the Waters XTerra^®^ RP8 column was selected as the optimal choice. Moreover, different column temperatures were tried to choose the optimum temperature, since key HPLC parameters such as viscosity, mobile phase polarity, and diffusivity are temperature-dependent, thus working at higher temperatures can enhance analysis conditions. This allows for a reduction in the organic solvent percentage in the mobile phase, leading to faster analysis without compromising efficiency^35^. The optimum temperature was found to be 40 °C. The final optimized conditions for the separation of IMG and MTF included a mobile phase consisting of 0.1% 1-octane sulfonic acid sodium salt monohydrate in purified water and ethanol (80:20, %*v/v*), a flow rate of 1.0 mL·min⁻¹, and a Waters XTerra^®^ RP8 column maintained at 40 °C (Fig. 2). The results of system suitability parameters of all studied chromatographic conditions are presented in Table 1. Chromatograms of IMG and MTF at different chromatographic parameters during method optimization are shown in Fig. S2 to S5.

**Table 1:** The system suitability parameters for all studied chromatographic conditions for optimizing the chromatographic separation of IMG and MTF.

Condition	t_*R*_ (min)		*N*		T		*R*_s_	k
	MTF	IMG	MTF	IMG	MTF	IMG		(IMG)
Type of ion pairing agent								
1-Hexane sulfonic acid sodium salt	2.5	2.8	6390	6391	1.0	0.99	2.72	0.64
1-Heptane sulfonic acid sodium salt	3.3	3.9	7635	7450	1.0	0.99	3.91	1.3
1-Octane sulfonic acid sodium salt	4.8	6.05	8313	8137	0.98	0.95	5.2	2.5
Type of organic modifier								
MeOH	5.7	9	9558	8845	0.9	0.88	10.4	4.3
ACN	3.3	4.3	9314	9672	1.0	1.0	6.1	1.3
EtOH	4.8	6.05	8313	8137	0.98	0.95	5.2	2.5
Type of stationary phase								
Hypersil BDS C8	7.2	9.2	6524	6578	1.4	1.54	4.8	4.1
Luna C8	9.6	12.7	12,128	11,597	1.1	1.05	7.6	6.3
XTerra RP8	4.8	6.05	8313	8137	0.98	0.95	5.2	2.5
Ethanol (organic phase) ratio								
15% EtOH	5.9	8.4	9182	8593	0.93	0.89	8.2	3.9
20% EtOH	4.8	6.05	8313	8137	0.98	0.95	5.2	2.5
25% EtOH	3.9	4.5	7615	7337	0.96	0.96	3.3	1.6
Effect of column temperatures								
30 ^0^C	5.1	6.5	8638	8716	0.97	0.96	5.7	2.8
40 ^0^C	4.8	6.05	8313	8137	0.98	0.95	5.2	2.5
50 ^0^C	4.6	5.8	6863	6342	0.89	0.89	4.53	2.4
Optimum condition								
Column: Waters XTerra RP8 (150 × 4.6 mm, 5 μm), mobile phase: 0.1% octane sulfonic acid : EtOH (80:20, %*v/v*), 40 °C	4.8	6.05	8313	8137	0.98	0.95	5.2	2.5

t_R_ :retention time, N: number of theoretical plates, T: USP tailing factor, R_s_: resolution, *k*: capacity factor, MeOH: methanol, EtOH: ethanol, ACN: acetonitrile.

### Optimization of stress conditions

**Fig. 3:**
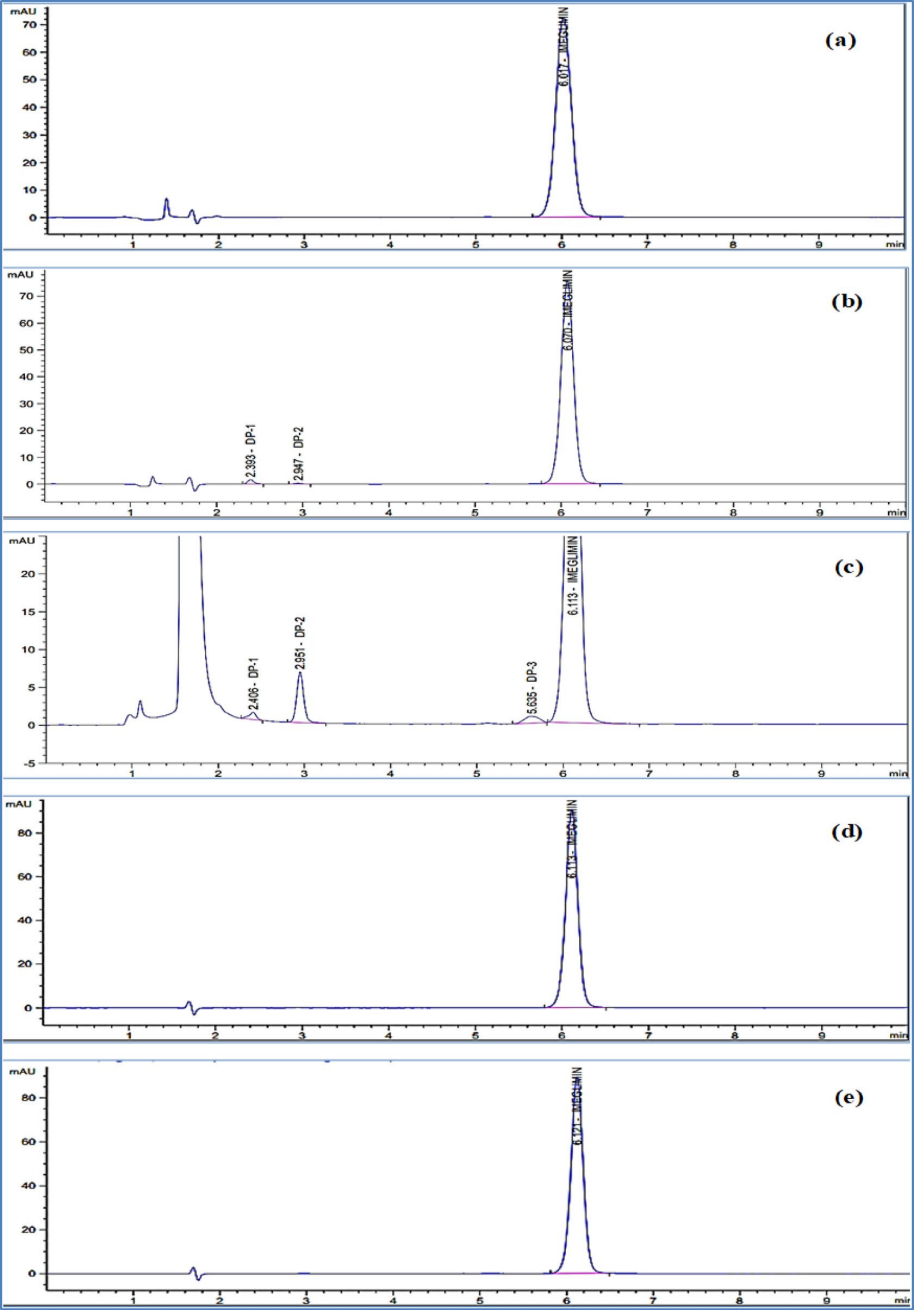
HPLC chromatograms of IMG subjected to various stress degradation conditions: (**a**) acidic hydrolysis; (**b**) alkaline hydrolysis; (**c**) oxidative degradation; (**d**) photolytic degradation; and (**e**) thermal degradation.

IMG was subjected to various stress degradation conditions, including photolysis, acidic and basic hydrolysis, thermal degradation, and oxidative degradation (Fig. 3). This study aimed to assess the intrinsic stability of IMG and elucidate its degradation mechanisms. Generally, 5–20% drug degradation is considered sufficient for chromatographic assay validation^36^. In this study, 10% degradation was established as appropriate for analysis, aligning with stability limits where approximately 90% of the labeled claim is acceptable.

The study design involved preparing two samples for each stress condition: a zero-time sample stored under normal conditions and a stressed sample exposed to degradation conditions. The results were compared to the unstressed drug, and the average percentage of degradation was calculated. Table S1 summarizes the tested stress conditions and corresponding degradation percentages.

#### Degradation results

IMG exhibited considerable stability under severe acidic conditions (5 M HCl), with no degradation detected, as confirmed by the absence of degradation peaks in chromatograms, peak purity analysis, and consistent peak area with the unstressed drug, which indicates its stability in acidic environments (Fig. 3a). Under optimized basic degradation conditions using 2 M NaOH, approximately 14% degradation of IMG was observed after 24 h. Two degradation peaks were detected at relative retention times (RRT) of 0.394 and 0.486 (Fig. 3b). Similarly, oxidative degradation using 30% hydrogen peroxide (H₂O₂) resulted in 12% degradation, with three degradation peaks appearing at RRT of 0.39 and 0.48—corresponding to the same degradants formed under basic conditions—and an additional peak at RRT of 0.92 (Fig. 3c). The similarity of the first two peaks suggests the formation of the same degradation products under both conditions^15^. The additional peak at RRT 0.92 is tentatively attributed to the formation of a nitro derivative^15^. The suggested basic and oxidative degradation pathways of IMG are illustrated in Fig. S6. Photo degradation studies confirmed that IMG remained stable under light exposure, as no degradation peaks were observed (Fig. 3d). Similarly, thermal degradation studies showed no degradation, confirming IMG’s stability under heat stress (Fig. 3e).

**Fig. 4:**
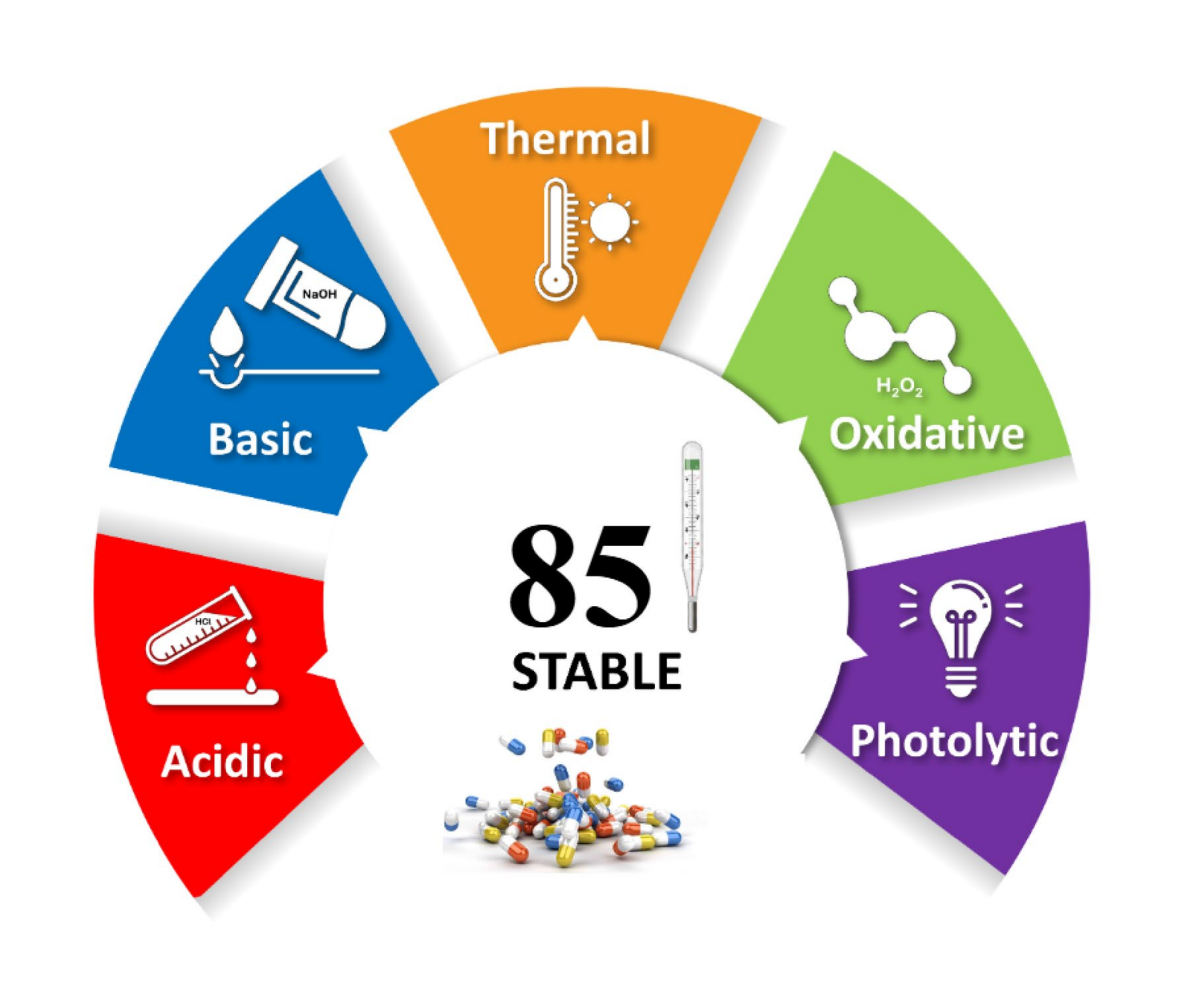
STABLE pictogram for IMG as a visual summary of its stability profile across five key stress conditions.

The Stability Toolkit for the Appraisal of Bio/Pharmaceuticals’ Level of Endurance (STABLE) was employed to provide a standardized, quantitative evaluation of IMG’s intrinsic stability under a suite of forced degradation conditions^37^. This holistic framework integrates experimental parameters from acid/base hydrolysis, thermal, oxidative, and photolytic stress testing into a unified scoring system, culminating in a total stability score and a corresponding visual pictogram. The application of STABLE to the experimental data obtained for IMG yielded a total score of 85. This score reflects a generally high level of intrinsic stability, though it reveals a refined profile of resilience across different stress vectors. The accompanying STABLE pictogram, presented in Fig. 4, offers an immediate visual summary of this profile. It indicates exceptional stability under acidic, thermal, and photolytic conditions, as evidenced by the absence of significant degradation. Conversely, the pictogram clearly highlights a low to moderate susceptibility to both basic and oxidative stress, consistent with the observed degradation of approximately 14% and 12%, respectively. This quantitative and visual output from the STABLE framework guides critical decisions in formulation development, particularly the preferred use of protective measures against alkaline pH and oxidizing agents and informing appropriate packaging and storage conditions to ensure product quality throughout IMG’s shelf life.

### Validation of the developed method

In accordance with ICH Q2 (R2) guidelines^38^, the chromatographic stability indicating method (SIAM) and dissolution method were validated with regard to specificity, repeatability, accuracy, linearity, and intermediate precision.

#### Linearity

For SIAM, it was found that the calibration graph was linear across the concentration range of 0.4 to 96.0 µg. mL^− 1^, and of 8.0 to 64.0 µg. mL^− 1^ for dissolution study. Statistical parameters for the developed methods including concentration ranges, correlation coefficients, linear regression equations, SD of the intercept (S_a_), and slope (S_b_), and SD of residuals (S_y/x_) were calculated and listed in Table 2. The linearity of IMG showed high value of the correlation coefficient and low value of intercept.

**Table 2:** Regression parameters for validation of SIAM and dissolution method.

Parameters	SIAM method	Dissolution method
Concentration range (µg. mL^− 1^)	0.4–96.0	8.0–64.0
Limit of detection (LOD) (µg. mL^− 1^)	0.224	0.352
Limit of quantitation (LOQ) (µg. mL^− 1^)	0.679	1.066
Regression parameters		
Slope ± SD (S_b_)	22.8879 ± 1.554	22.9922 ± 0.061
Intercept ± SD (S_a_)	-5.0666 ± 0.039	-7.05264 ± 2.451
SD of residuals (S_y/x_)	66.738	59.355
Correlation coefficient (r)	0.99998	0.999958

#### Limit of quantitation and limit of detection

The following equations were used to calculate LOQ and LOD:

$$LOD{\text{ }} = {\text{ }}3.3\,{\sigma \mathord{\left/ {\vphantom {\sigma S}} \right. \kern-\nulldelimiterspace} S}$$

$$LOQ{\text{ }} = 10\,{\sigma \mathord{\left/ {\vphantom {\sigma S}} \right. \kern-\nulldelimiterspace} S}$$

Where S is the slope of the calibration curve, and σ is the standard deviation of the intercept. The LOD and LOQ values as listed in Table 2 were 0.224 µg. mL^− 1^ and 0.679 µg. mL^− 1^, respectively for SIAM, and were 0.352 µg. mL^− 1^ and 1.066 µg. mL^− 1^, respectively for dissolution study which indicate the method’s sensitivity.

#### Accuracy

The SIAM method was applied for the quantitation of both IMG drug substance and IMG in spiked placebo over the studied concentration range. The accuracy was evaluated by calculating the mean % recovery ± SD for three concentrations covering the linearity range, as shown in Table 3. For dissolution test, the accuracy was prepared in triplicate using the standard addition method (enriched placebo). According to ICH Q2 (R2), the accuracy was started from 25% to cover the lowest ranges (Q-45)^28^. The mean % recovery was calculated and listed in Table 3.

**Table 3:** Results of accuracy studies of IMG assay and dissolution testing.

Method	Sample	Conc. taken (µg. mL^− 1^)	Mean conc. found* (µg. mL^− 1^)	% Recovery	Mean % recovery ± SD
Assay (SIAM)	Drug substance	30.0	29.95	99.83	99.65 ± 0.36
		36.0	35.96	99.89	
		50.0	49.62	99.24	
	Spiked placebo	32.0	32.25	100.77	100.3 ± 0.73
		40.0	40.27	100.67	
		48.0	47.74	99.46	
Dissolution	Spiked placebo	10.0	9.925	99.25	100.08 ± 0.933
		40.0	39.963	99.91	
		48.0	48.522	101.09	

*Average of three determinations.

#### Precision

Repeatability (intra-day) was assessed over the concentration range through 3 replicates analysis over 3 concentrations of IMG drug substance and 6 determinations at 100% concentration from tablet dosage form. Intermediate precision (inter-day) was tested by repeated analysis of IMG drug substance and in tablet dosage form using the selected concentrations for two successive days. The precision of the SIAM method was confirmed by low % pooled RSD as shown in Tables 4 and 5. The precision for dissolution study was investigated on six Twymeeg^®^ 500 mg FCT. They were exposed to the optimized dissolution conditions. The intra-day precision was evaluated with the relative standard deviation (RSD) of the six determinations. The intermediate precision (inter-day) was determined by evaluating the results of two different days. The results are listed in Table 5.

**Table 4:** Results of precision study for assay of IMG drug substance.

Conc. taken (µg. mL^− 1^)	Intra-day				Inter-day				Pooled RSD
	Conc. found (µg. mL^− 1^)	Mean* % recovery	SD	RSD (%)	Conc. found (µg. mL^− 1^)	Mean* % Recovery	SD	RSD (%)	
75.0	74.873	99.83	0.07	0.07	75.76	101.02	0.37	0.36	0.09
90.0	89.901	99.89	0.30	0.30	90.45	100.50	0.23	0.23	
125.0	124.05	99.24	0.12	0.12	125.05	100.04	0.31	0.31	

*Average of three determinations.

**Table 5:** Results of precision study for assay and dissolution of IMG in tablets dosage form.

Method	Conc. taken (µg. mL^− 1^)	Intra-day precision				Inter-day precision				Pooled RSD
		Conc. found (µg. mL^− 1^)	Mean* conc. found (µg. mL^− 1^)	SD	RSD (%)	Conc. found (µg. mL^− 1^)	Mean*conc. found (µg. mL^− 1^)	SD	RSD (%)	
Assay	40.0	39.61	39.89	0.23	0.58	40.38	40.24	0.35	0.86	0.835
	40.0	40.23				40.34				
	40.0	39.87				40.31				
	40.0	40.10				40.40				
	40.0	39.81				39.55				
	40.0	39.74				40.49				
Dissolution	40.0 40.0 40.0 40.0 40.0 40.0	39.31 39.27 39.31 39.25 39.30 39.25	39.28	0.03	0.07	40.32 40.22 40.26 40.34 40.32 40.38	40.31	0.06	0.15	1.35

*Average of six determinations.

#### Specificity and interference

**Fig. 5:**
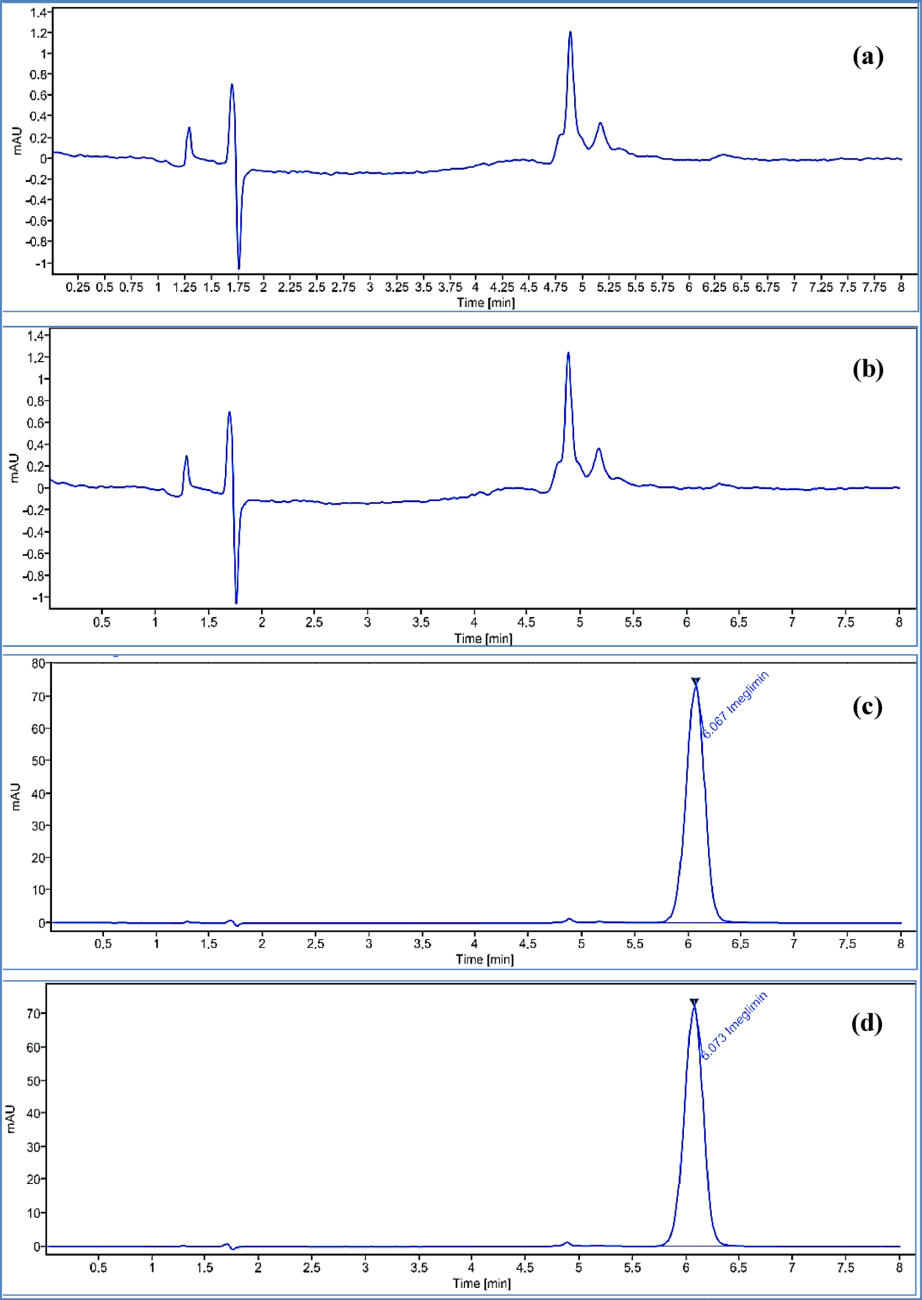
HPLC chromatograms of (**a**) dissolution media (0.1 M HCl), (**b**) placebo, (**c**) standard dissolution, and (**d**) test dissolution of IMG.

The developed method demonstrated high specificity, showing no interference from degradation products, MTF, or excipients. This was confirmed by peak purity analysis under various stress conditions which indicated the main IMG peak remained spectrally pure and well-resolved from all degradation peaks Fig. S7 to S11. Moreover, MTF was chromatographically separated and eluted at a distinct retention time, confirming no overlap with the IMG peak (Fig. 2b). Additionally, neither the dissolution medium nor the tablet placebo exhibited any interfering peaks at the retention time of IMG (Fig. 5).

#### Robustness

The procedure robustness was tested through introducing minor changes in the method parameters including the changes in the temperature of the column (40 ± 2 °C), and in the flow rate (1.00 ± 0.05). The method remains unaffected by small and deliberate changes in chromatographic parameters and provides an indication of the suitability of the method within normal usage. The pooled RSD of the results is found to be less than 3%.The results for assay and dissolution analyses are listed in Table 6.

**Table 6:** The results of robustness study of IMG assay and dissolution.

Method	Condition	Conc. taken (µg. mL^− 1^)	T	*N*	Mean** % recovery	SD	RSD (%)	Pooled RSD (%)
Flow rate (mL. min^− 1^)								
Assay	0.95 1.00* 1.05	40.0	0.9734 0.9762 0.9798	5826 5686 5488	98.81 99.77 98.68	0.43 0.78 0.45	0.435 0.784 0.453	0.72
Dissolution	0.95 1.00* 1.05	40.0	0.948 0.956 0.951	5234 5440 5185	99.26 99.28 99.00	0.123 0.096 0.047	0.124 0.097 0.047	0.16
Column temperature (°C)								
Assay	38 40* 42	40.0	0.9718 0.9732 0.9741	5552 5686 5466	99.38 99.77 98.90	0.402 0.782 0.449	0.405 0.784 0.454	0.63
Dissolution	38 40* 42	40.0	0.954 0.956 0.932	5466 5440 5033	99.20 99.28 98.92	0.142 0.096 0.112	0.143 0.097 0.113	0.19

*Optimum chromatographic conditions, **Average of three determinations, T: USP tailing Factor, N: number of theoretical.

plates.

#### Stability of solution

By comparing the responses of two standard IMG solutions made from two different stock IMG solutions, the stability of the IMG stock solution was evaluated after 48 h. The first solution was freshly prepared and the second was diluted from the stock prepared on the previous day and injected after 48 of preparation. The stock solutions were found to be stable and can be used for sample preparation for more than 24 h. The results for SIAM and dissolution method are shown in Table 7.

**Table 7:** Stability of IMG assay and dissolution stock standard solutions.

Method	Standard stock solution	Conc. taken (µg. mL^− 1^)	Mean Conc. found* (µg. mL^− 1^)	Mean % recovery *	RSD (%)
Assay	Freshly prepared	40.0	40.108	100.27	0.192
	After 48 h. of preparation	40.0	40.376	100.94	0.122
Dissolution	Freshly prepared	40.0	39.84	99.60	0.218
	After 48 h. of preparation	40.0	40.292	100.73	0.144

*Average of six determination.

## Application in dissolution testing

The method was effectively applied for dissolution testing without any interference from dissolution media, placebo, or test samples with the peak of IMG. The dissolution profile of Twymeeg^®^ 500 mg tablets in different pH-media was studied and the results are listed in (Table 8). The results showed a pH-dependent dissolution profile, with the most rapid and complete release occurring in the 0.1 M HCl medium, where over 99% of the drug was dissolved within 45 min. In contrast, the dissolution was slower in the pH 4.50 and pH 6.80 media, with final percentages of 96.2% and 99.5%, respectively. These results indicated that the developed method was sufficiently reliable for dissolution testing of IMG.

**Table 8:** Percentage dissolved of IMG tablets (Twymeeg^®^ 500 mg) in different dissolution media.

Media	Average* (%) dissolved per media					
	5 min	10 min	15 min	20 min	30 min	45 min
0.1 M HCl	31.3	62.8	88.1	97.1	99.6	99.8
pH 4.50	29.1	58.0	80.0	89.2	89.6	96.2
pH 6.80	29.4	53.8	69.7	84.7	99.3	99.5

*Average of six determinations. A volume of 500 mL of each media was used, rotation speed was set at 50 RPM, temperature adjusted at 37 °C.

## Assessment of the method greenness

**Fig. 6:**
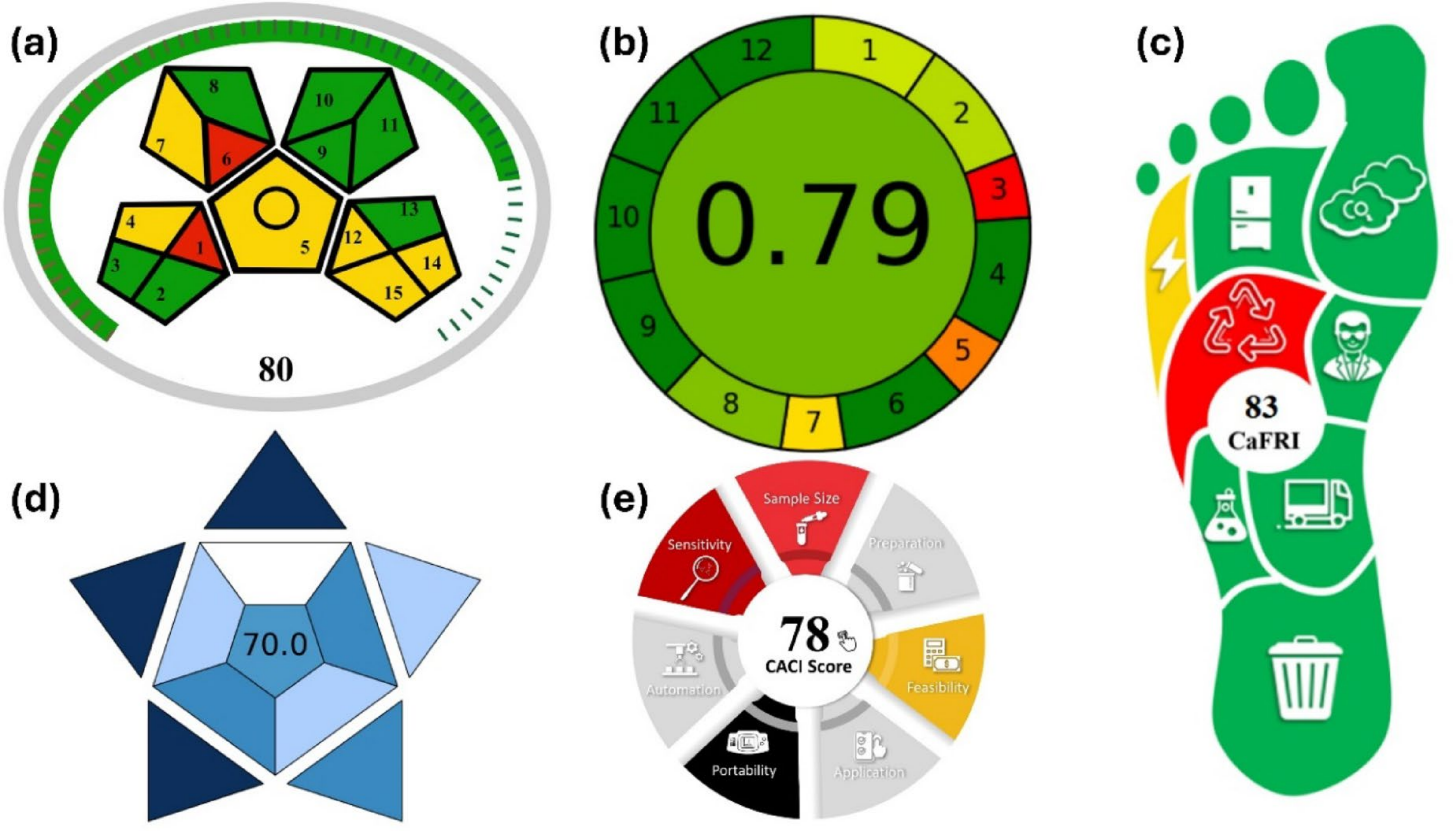
Assessment of the developed method using (**a**) MoGAPI, (**b**) AGREE, (**c**) CaFRI, (**d**) BAGI and (**e**) CACI.

Greenness assessment of analytical methods is an important aspect of green analytical chemistry, which focuses on development of eco-friendly and safer analytical methods. The Analytical Eco-Scale was employed to evaluate the greenness of the developed method (Table 9). It is a semi-quantitative tool used to assess the environmental friendliness of analytical procedures. It operates by subtracting penalty points (PPs) from a total score of 100 for each parameter that does not meet the ideal conditions of green analysis. A score below 50 indicates that the method is inadequately green, a score between 50 and 75 suggests acceptable greenness, and a score above 75 reflects excellent greenness^39–41^. The developed method achieved a total score of 81, demonstrating greenness of the developed method. In addition to the Analytical Eco-Scale, the greenness of the developed RP-HPLC method was further assessed using two widely accepted green analytical chemistry tools: the Analytical GREEnness metric (AGREE)^42^ and the modified Green Analytical Procedure Index (MoGAPI)^43,44^. As shown in Fig. 6a, the MoGAPI pictogram integrates both qualitative and quantitative aspects of method greenness across five main criteria: sample preparation, reagents, instrumentation, waste, and occupational hazards. MoGAPI confirmed the high greenness profile of the method, with a total score of 80. The favorable MoGAPI score highlights the minimal use of toxic reagents, the low environmental burden of ethanol as a mobile phase component, and the suitability of the method for routine use with reduced health and environmental impact. Furthermore, AGREE, which evaluates compliance with the 12 principles of green analytical chemistry, generated a holistic visual representation (Fig. 6b) confirming that the method aligns well with green principles, particularly in its choice of environmentally benign solvents (ethanol) and minimized waste generation. These complementary assessments reinforce the environmental sustainability of the developed method and its suitability for implementation in green quality control laboratories.

**Table 9:** Calculation of the analytical Eco-Scale score for the developed RP-HPLC method for IMG assay.

Parameters				Penalty Points (PPs)
Reagents				
Type	Hazard	Amount	Subtotal PPs	
Ethanol	two pictograms, danger	10–100 mL	4*2	8
Octane sulfonic acid	one pictogram, danger	1 mL	2*1	2
Total PPs for reagents (∑ hazard* amount)				10
Instrument				
Energy consumption		≤ 1.5 kW/h (1)		1
Emission of vapors or gasses		none		0
Waste				
Waste generated		> 10 mL		5
Waste treatment		No treatment		3
Total PPs for waste				8
Total PPs				19
Analytical Eco-Scale Score (= 100-PPs)				81

To provide a comprehensive, quantitative and multi-faceted evaluation, the method was also assessed using the Carbon Footprint of Analytical Methods for Relative Environmental Impact (CaFRI) tool^45^, as shown in Fig. 6c. The CaFRI assessment corroborated the excellent environmental profile, highlighting advantages such as low energy consumption due to the analysis time of less than 10 min, a low carbon footprint from instrument power usage, the absence of nitrogen gas, minimal waste generation (< 10 mL per sample), and the use of a semi-automatic system requiring only one operator. These complementary assessments reinforce the environmental sustainability of the developed method and its suitability for implementation in green quality control laboratories.

To evaluate the method’s practicality and economic feasibility which are key aspects of sustainable method development, the blueness procedure was assessed. The Blue Applicability Grade Index (BAGI)^46^ was used to evaluate practical attributes such as analysis time, easiness, and operational complexity (Fig. 6d). The high BAGI score confirmed the method’s excellent practicality and user-friendliness, making it ideally suited for high-throughput routine analysis. Furthermore, the method was evaluated using the Click Analytical Chemistry Index (CACI)^47^, a tool designed to assess the practicality and cost-efficiency of analytical procedures (Fig. 6e). The high CACI scores highlighted the method’s straightforward implementation and high potential for successful adoption in quality control laboratories.

## Comparison with other reported methods

The developed method was compared with previously published methods in terms of chromatographic conditions, retention time, sensitivity, and Eco-Scale total score )Table 10(. It demonstrated superior sensitivity and comparable greenness, along with an acceptable retention time that enables the detection of all potential impurities and degradants. Additionally, the method achieved a low LOQ of 0.679 µg. mL^− 1^, making it applicable for the detection of IMG in plasma since the reported C_max_ of IMG in plasma is approximately 1000 ng. mL^− 1^ following oral administration of a 500–600 mg dose^48^.

**Table 10:** Comparison between the developed RP-HPLC method and previously reported LC methods for the determination of IMG.

Chromatographic conditions			Retention time (min)	Sensitivity (Linearity range, µg/mL)	LOD (µg/mL)	LOQ (µg/mL)	Analytical Eco-scale total score	Reference
Mobile phase	Stationary phase	Detection λ (nm)						
Methanol: 10mM phosphate buffer (pH 6.0) (80:20, *% v/v*)	BRISA LC2 C18 (250 × 4.6 mm, 5 μm)	243	3.097	0.1–30	0.137	0.417	79	^12^
Buffer (pH 3.0): methanol (75:25, *% v/v*), with orthophosphoric acid to adjust pH	Hypersil ODS (150 × 4.6 mm, 5 μm)	234	6.3	100–300	Not mentioned	Not mentioned	79	^13^
Phosphate buffer (pH 4.2): acetonitrile (80:20, *% v/v*)	Credchrom C18 column (250 × 4.6 mm, 5 μm)	241	2.5	5–50	0.578	1.751	83	^7^
Methanol: 0.1% orthophosphoric acid (Triethylamine added to adjust pH to 4.2) (40:60, *% v/v)*	C18 AGILENT	240	4.7	10–50	0.258	0.783	75	^11^
0.1%octane sulfonic acid sodium salt in water: Ethanol (80:20, *% v/v*)	XTerra RP8 (150 × 4.6, 5 μm)	241	6.0	0.4–96	0.224	0.679	81	This work

## Conclusion

In conclusion, the developed RP-HPLC method was successfully applied for the determination of IMG in pharmaceutical tablets and in the presence of its common impurity, metformin. It also proved effective in separating IMG from all potential degradation products under different stress conditions. The method was validated according to ICH guidelines, demonstrating satisfactory results for sensitivity, specificity, accuracy, precision, linearity, range, and robustness. Furthermore, it was efficiently utilized to evaluate the in vitro dissolution profile of IMG in its commercial dosage form. Unlike previously reported RP-HPLC methods, the proposed method is environmentally friendly, employing ethanol as a green solvent and octane sulfonic acid as an ion-pairing reagent. This ion-pairing approach overcomes the limitation of low retention times in previously reported stability-indicating methods, enabling improved resolution and detection of impurities and degradants. Overall, the method is suitable for routine quality control, dissolution testing, and has potential applicability for the determination of IMG in biological matrices such as plasma.

## Supplementary Information

Below is the link to the electronic supplementary material.

Supplementary Material 1

## Data Availability

All data generated or analysed during this study are included in this published article [and its supplementary information files].
