# Supplementary material for: Stability indicating green HPLC method for Imeglimin hydrochloride determination in pharmaceutical tablets with comprehensive assessment
Source: Sci Rep. 2026 Jan 10;16:1334. doi: 10.1038/s41598-025-32999-4 (PMC12796370; doi:10.1038/s41598-025-32999-4)
Supplement: Supplementary file 1 — Supplementary Material 1 [file 41598_2025_32999_MOESM1_ESM.docx]

**Stability Indicating Green HPLC Method for Imeglimin Hydrochloride Determination in Pharmaceutical Tablets with Comprehensive Assessment**

**Supplementary Information**

**Table S1:** The percentage of degradation in different degradation conditions.

| **Stress condition** | **Degradation %** |
| --- | --- |
| **Acid degradation**  2.0 mL of 1M HCl 12h, reflux  2.0 mL of 3M HCl 24h, reflux  2.0 mL of 5M HCl 24h, reflux | 0.00  0.00  0.00 |
| **Base degradation**  2 mL of 1M NaOH 12h, reflux  2.0 mL of 2M NaOH, reflux for 24h *  2.0 mL of 5M NaOH, reflux for 24h | 2.00  14.00  30.00 |
| **Photo degradation**  6 million lux hours | 0.00 |
| **Oxidative degradation**  2.0 mL of 30% H_2_O_2_, at room temperature for 24 h  2.0 mL of 30% H_2_O_2_, reflux for 24 h *  3.0 mL of 30% H_2_O_2_, reflux for 24 h | 0.00  12.00  17.00 |
| **Heat Degradation**  360 min, at 80 ^0^C | 0.00 |

*: optimum degradation conditions.


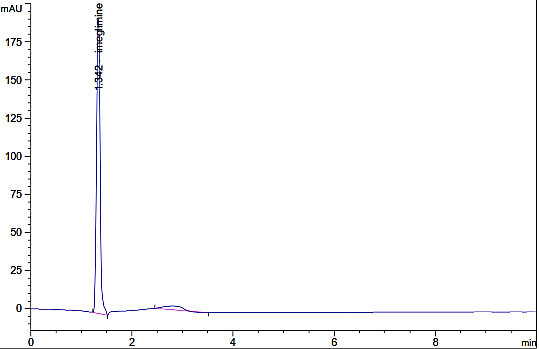


**Fig. S1**: HPLC chromatogram of IMG (40.0 µg. mL^-1^) using a mobile phase consisting of purified water : EtOH (80:20, % *v/v*) on XTerra RP8 column, at column temperature 40 ^°^C, flow rate 1.0 mL. min^-1^, and injection volume 5µL.

**Fig. S2**: HPLC chromatogram of IMG (40.0 µg. mL^-1^) and MTF (4.0 µg. mL^-1^), using different organic modifiers a) Acetonitrile, b) Ethanol, and c) Methanol on XTerra RP8 column, mobile phase consists of 0.1% of 1-Octane sulfonic acid sodium salt : organic modifier (80:20, % *v/v*), at column temperature 40 ^°^C, flow rate 1.0 mL.min^-1^, and injection volume 5µL.

**Fig. S3**: HPLC chromatogram of IMG (40.0 µg. mL^-1^) and MTF (4.0 µg. mL^-1^), using different ion pairing agent in mobile phase a) 0.1% of 1- hexane, b) 0.1% of 1-heptane, and c) 0.1% of 1-octane sulfonic acid sodium salt on XTerra RP8 column, mobile phase consists of ion pairing agent : EtOH (80:20, % *v/v*), at column temperature 40 ^°^C, flow rate 1.0 mL.min^-1^, and injection volume 5µL.

**Fig. S4:** HPLC chromatogram of IMG (40.0 µg. mL^-1^) and MTF (4.0 µg. mL^-1^), using different ratios of EtOH in mobile phase a) 15%, b) 20%, and c) 25% *v/v* of EtOH on XTerra RP8 column, mobile phase consists of 0.1% of 1-octane sulfonic acid sodium salt: EtOH, at column temperature 40 ^°^C, flow rate 1.0 mL.min^-1^, and injection volume 5µL.

**Fig. S5**: HPLC chromatogram of IMG (40.0 µg. mL^-1^) and MTF (4.0 µg. mL^-1^), using different column temperature a) 30 ^°^C, b) 40 ^°^C, and c) 50 ^°^C on XTerra RP8 5 µm, (4.6 x 150) mm column, mobile phase consists of 0.1% of 1-octane sulfonic acid sodium salt: EtOH (80:20, % *v/v*), flow rate 1.0 mL.min^-1^, and injection volume 5µL.


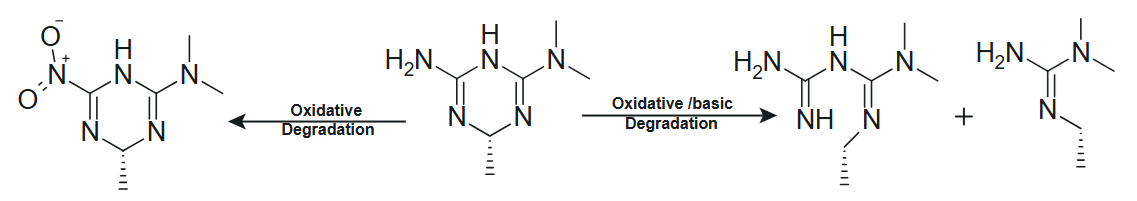


**Fig. S6**: The suggested pathway for alkaline and oxidative degradation of IMG.

**
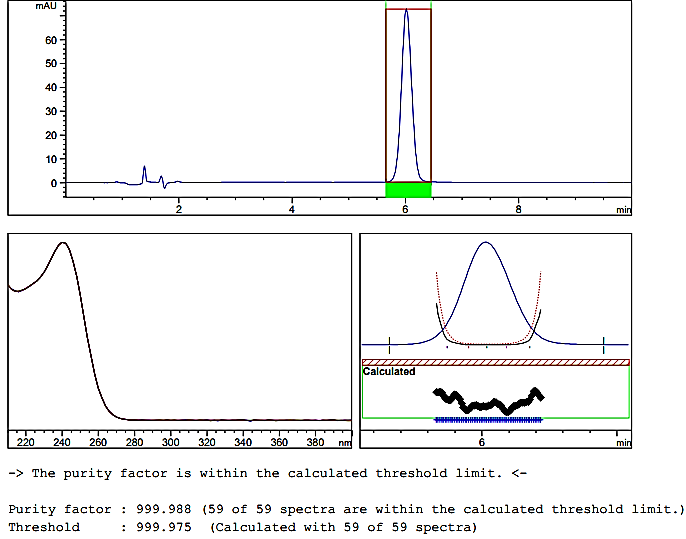
Fig. S7:** HPLC peak purity of acidic degradation.


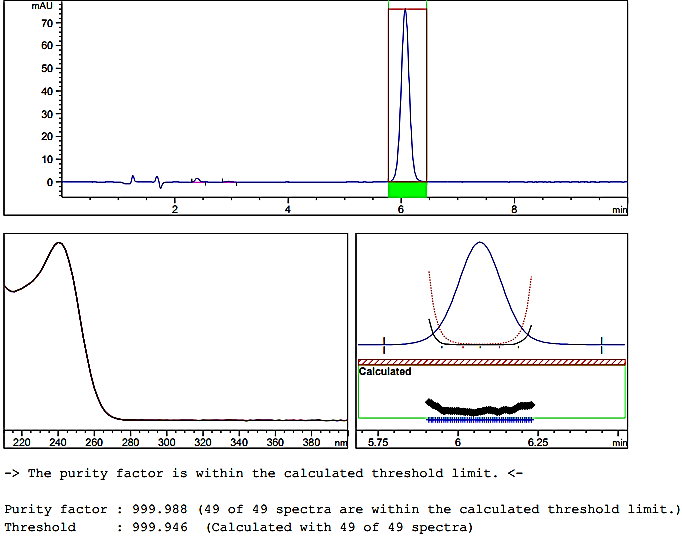


**Fig. S8:** HPLC peak purity of alkaline degradation.


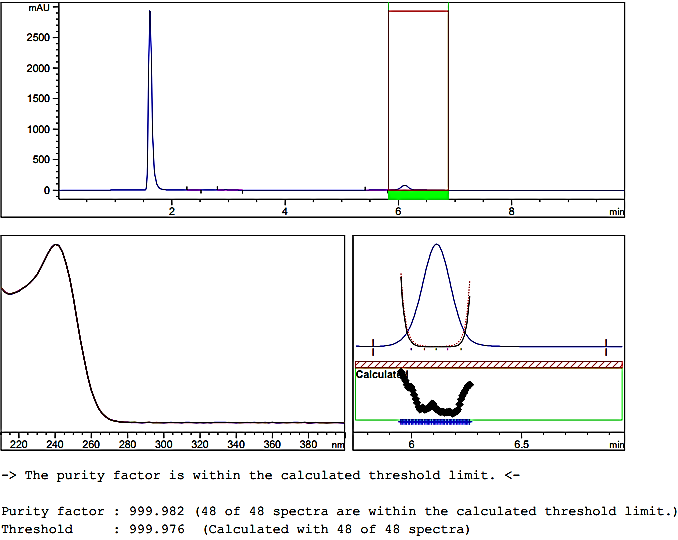
**Fig. S9:** HPLC peak purity of oxidative degradation.


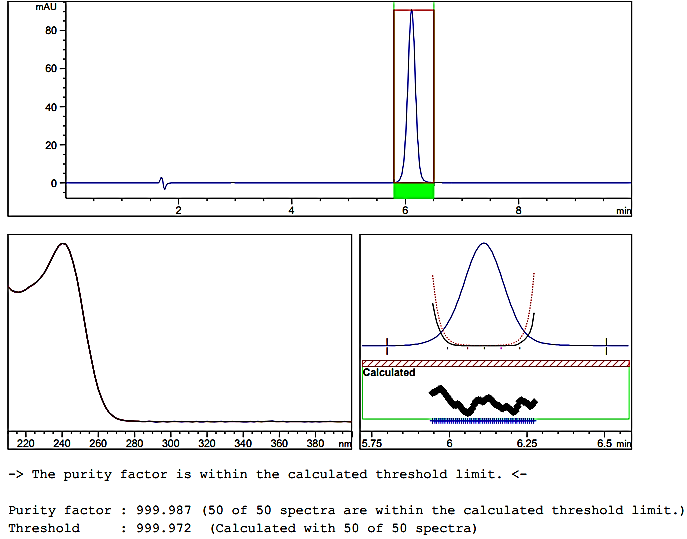


**Fig. S10:** HPLC peak purity of photo degradation.


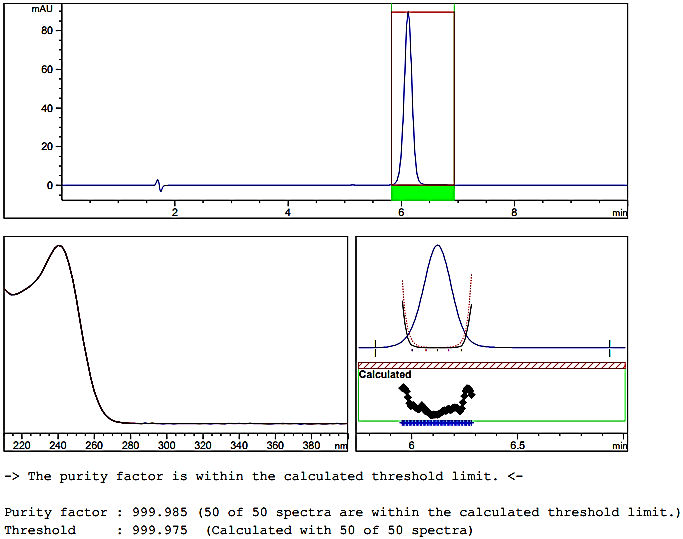


**Fig. S11:** HPLC peak purity of thermal degradation.
